# Improving primary care access for rural women Veterans: the Boost Team

**DOI:** 10.3389/frhs.2024.1149086

**Published:** 2024-07-08

**Authors:** Jenny K. Cohen, Lindsey L. Monteith, Tara Stacker, Michaela McCarthy, Mayan Bomsztyk, Abigail Wilson, Jennifer Childers, Tanvir Hussain, Jeffrey Kohlwes

**Affiliations:** ^1^ United States Department of Veterans Affairs, San Francisco VHA Health Care System, Veterans Health Administration, San Francisco, CA, United States; ^2^ Department of Medicine, School of Medicine, University of California San Francisco, San Francisco, CA, United States; ^3^ VA Rocky Mountain Mental Illness Research, Education and Clinical Center for Suicide Prevention, SAurora, CO, United States; ^4^ Department of Physical Medicine and Rehabilitation, University of Colorado Anschutz Medical Campus, SAurora, CO, United States; ^5^ Denver-Seattle Center of Innovation for Veteran-Centered and Value-Driven Care (COIN), VA Eastern Colorado Health Care System, SAurora, CO, United States; ^6^ United States Department of Veterans Affairs, VHA Sierra Pacific Network (VISN 21), Veterans Health Administration, Mare Island, CA, United States

**Keywords:** Veterans Health Administration (VHA), rural health, gender diverse veterans, women veterans, outreach, primary care redesign, implementation science

## Abstract

**Objectives:**

To improve healthcare access for rural cisgender women and gender diverse Veterans, we created the “Boost Team,” a clinician-driven telehealth outreach service to connect this population to Veterans Health Administration (VHA) services.

**Methods:**

Between 9/2021 and 2/2022, we conducted a needs assessment in the Veterans Integrated Service Network (VISN) 21 and used those data to develop an outreach intervention. We piloted a clinician-led outreach intervention in 3/2022, and formally deployed an outreach team in 9/2022.

**Results:**

The needs assessment uncovered opportunities to educate Veterans, staff, and clinicians about available VHA women's health services, and a need for easily-accessible gender-sensitive services. During the pilot, 58% (7/12) rural cisgender women Veterans were successfully contacted, all reported positive experiences with the intervention. The formal outreach team launched in 9/2022 and consists of a nurse practitioner (NP), scheduler, Peer Support Specialist, and medical director. From 9/2022 to 12/2022 the Boost NP called 110 rural cisgender women and gender diverse Veterans and spoke to 65 (59%) of them. Common care needs identified and addressed included care coordination, new referrals, medication management, and diagnostics.

**Discussion:**

Data from Boost show that clinician-led outreach can engage rural cisgender women and gender diverse Veterans in VHA services, there is a desire for more gender-sensitive services, and there is a need for systems-level improvements to allow for improved care coordination and decreased leakage outside of VHA. Using robust strategies grounded in implementation sciences, we will continue conducting a program evaluation to study the impact of Boost and scale and expand the program.

## Introduction

It is estimated that women Veterans will represent 18% of the Veteran population by 2040. Unfortunately, there are concerns regarding timely, accessible, high-quality, gender-specific health services for women Veterans (1, 2). Such concerns may be further exacerbated among rural women Veterans, and may be particularly relevant in specific areas, such as Veterans Integrated Service Network (VISN) 21, where, as of 2018, only one-third of women Veterans were enrolled in Veterans Health Administration (VHA) healthcare (2). A 2019 systematic review showed evidence that patient navigation through telephonic outreach increases colorectal, breast, and cervical cancer screening among populations facing health disparities (3). Subsequent studies from the mental health and primary care fields have reported that telephonic clinician-driven outreach can result in more robust and meaningful care engagement than letters or standard in-person office visits (4, 5). Our work builds on existing literature from the mental health and primary care domains and adds novel data supporting the potential of clinician-driven outreach to improve access and utilization of care, specifically for rural cisgender women and gender diverse Veterans.

The Reach, Effectiveness, Adoption, Implementation, and Maintenance framework (RE-AIM) is commonly applied in implementation science to guide planning and deployment of health service interventions (6, 7). Its use in clinical contexts has evolved toward a practical approach that allows stakeholders to focus on select RE-AIM dimensions with iterative adjustments to make operational improvements (7). We used the RE-AIM framework to guide the development and analysis of an intervention involving clinician-driven telephone outreach program to cisgender women and gender diverse Veterans within one VHA facility.

This manuscript describes a Veteran-centered approach to creating a novel outreach and healthcare delivery program aimed at improving utilization of VHA services for rural cisgender women and gender diverse Veterans.

## Methods

### Design

Using the “Reach” dimension of Re-AIM, we sought to ascertain attitudes and perceptions about VHA among those involved with administering, facilitating, and consuming VHA services, to identify barriers and facilitators of VHA care utilization and opportunities for improvement (6, 7). Gaps identified through the needs assessment then informed project design, implementation, and quality improvement.

### Participants

**Figure 1:**
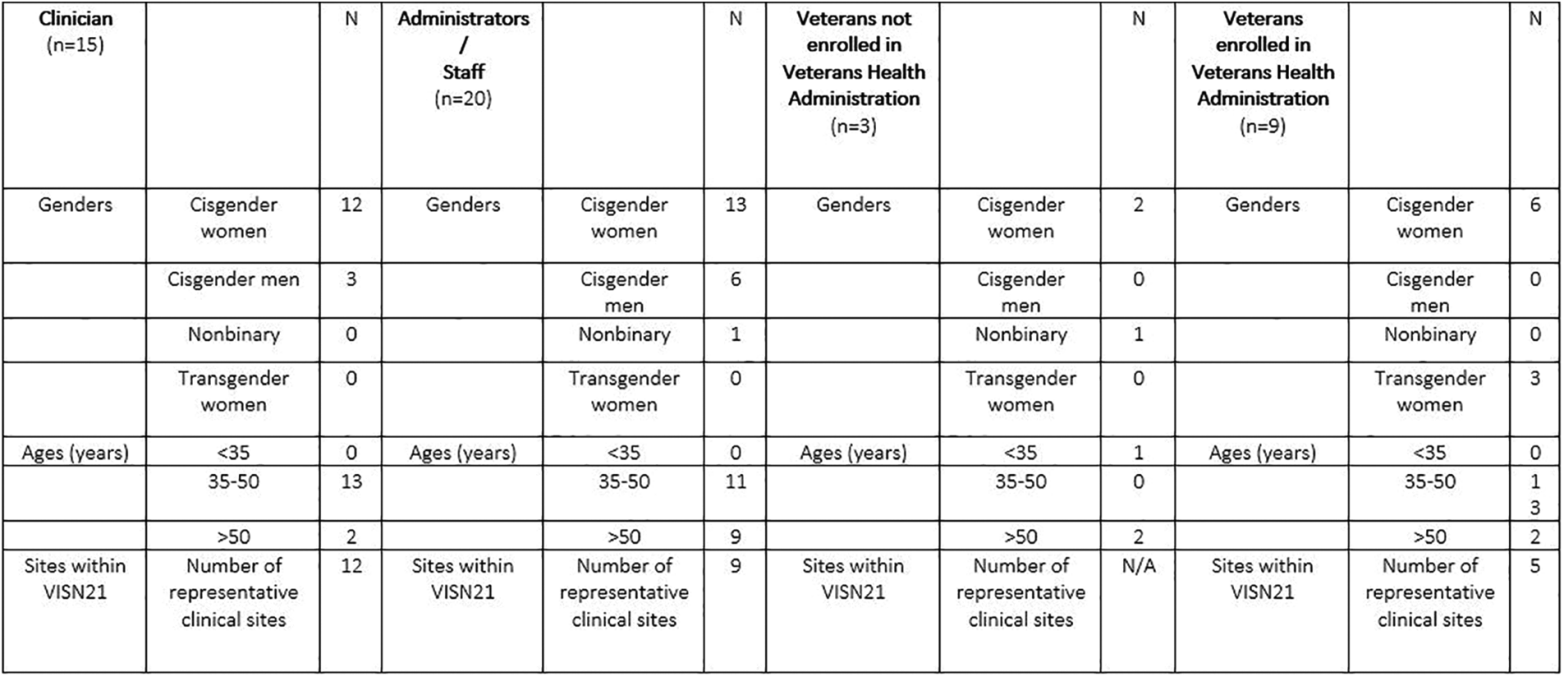
Demographics of needs assessment interviewees (*n* = 47).

Between September 2021 and February 2022, we interviewed staff/administrators (*n* = 20), clinicians (*n* = 15), and cisgender women and gender diverse Veterans (*n* = 12) in VHA rural community-based outpatient clinics (CBOCs) and VHA urban medical centers that serve as referral hubs for rural clinics in VISN 21 (Figure 1). Interview participants were identified through social snowballing techniques. Representative CBOCs included Yuba City, Eureka, Ukiah, Redding, and the San Francisco Women's Clinic in California, as well as Pahrump, Reno, and Carson City in Nevada. Three Veterans not currently enrolled in VHA (2 cisgender women; one Nonbinary Veteran) were interviewed. Of the three Veterans not enrolled in VHA, one did not qualify and two had previously been enrolled but left due to dissatisfaction with the system. Interviews occurred by phone or videoconferencing. Interviewees were informed about the concept and goals of the clinician-driven outreach intervention and asked a single question, “What should we know about your experiences with the VHA in order to try to make this work successful?” One individual conducted all interviews. Interviews were opt-out and part of routine quality improvement work. De-identified notes were taken during the interviews and the Precede-Proceed Framework was used to organize themes that emerged from the interviews (8).

### Materials

Findings from the needs assessment informed the creation of an outreach playbook and the formation of a pilot outreach team. The playbook contained a call script with introductory language about the outreach program; a framework for using motivational interviewing skills to elicit Veteran-centered wellness goals to uncover barriers and unmet needs; a reference guide of VHA resources; a roster of local CBOC staff and clinicians; and a list of regional community resources.

### Procedures

The needs assessment also informed the creation of an intervention prototype called the Boost Team (whose goal is to boost and elevate rural cisgender women and gender diverse Veteran health), comprised of a Medical Service Administrator (MSA), Nurse Practitioner (NP), Veteran Peer Support Specialist, and Physician Medical Director. In collaboration with VISN 21 Informatics and using data from the VHA Corporate Data Warehouse (CDW), we created an outreach dashboard of cisgender women and gender diverse Veterans enrolled in VHA and assigned to the San Francisco Station, and whose address mapped closest to the partnering rural CBOC. The Boost NP used the CDW-generated outreach dashboard to call rural cisgender women and gender diverse Veterans and provided clinical care, education on available resources, and assistance obtaining primary care with a designated women's health clinician within the VHA. The Boost Team tried to repatriate Veterans to their local CBOCs and coordinate follow-up with VHA primary care clinician, or to maximize Veteran healthcare choice, identified a different VHA clinician or clinic if that was the Veteran's preference. Local Patient Care-Aligned Care Teams (PACTs) were utilized in a collaborative manner to assist with care coordination and follow-up care.

Based on RE-AIM, we next focused on the “effectiveness” domain to guide the creation and analysis of a pragmatic trial evaluating the Boost intervention based at one partner rural CBOC (6, 7). In March 2022, we piloted the feasibility and acceptability of the Boost Team intervention, calling 12 rural cisgender women Veterans who had not been seen in VHA primary care for over a year and were randomly selected from the outreach dashboard. During each outreach call, the clinician, a second-year NP resident, reviewed the Veteran's electronic health record to identify actionable care gaps, used motivational interviewing to elicit wellness goals, and provided real-time clinical care.

Using the findings from the feasibility and acceptability pilot, we developed a Plan-Do-Study-Act (PDSA) Cycle 1 plan to implement and study a full-scale outreach intervention starting in September 2022. During this time, a dedicated Boost NP called cisgender women and gender diverse Veterans identified by the outreach dashboard and provided real-time clinical care and assistance with care coordination. The Boost NP attempted to call all Veterans on the dashboard. If the Boost NP was unable to reach a Veteran, a generic message regarding the Boost Team and the purpose of the outreach call was left on voicemail that included a call-back number directly to the Boost NP's telephone extension. The message did not include any Veteran identifying information or health information.

### Data collection and analysis

**Figure 2:**
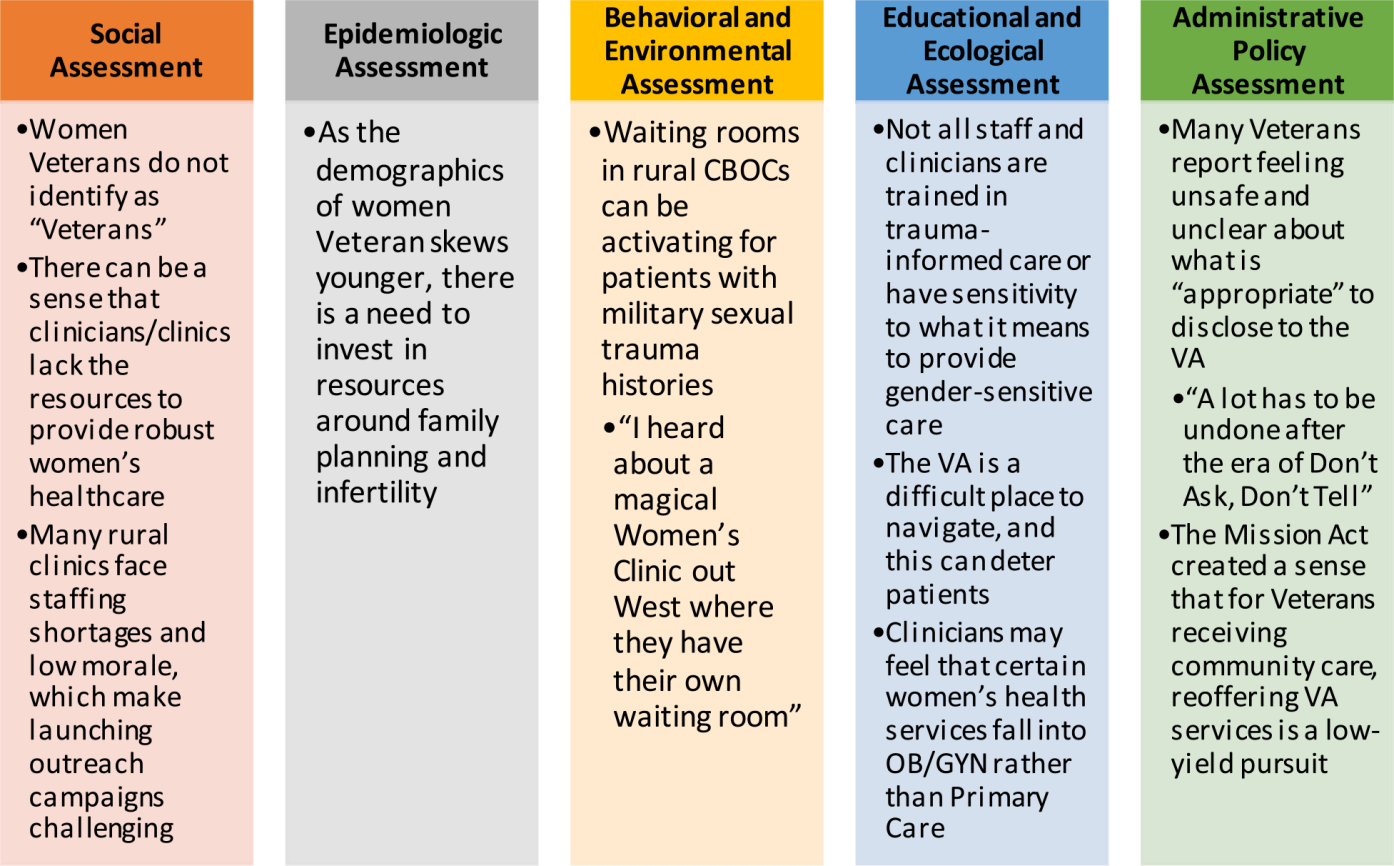
Lessons from the field: salient themes from the needs assessment.

Clinical care was documented in the electronic health record, workload credits were allocated, and patients did not have a co-pay for the phone encounter. Additionally, the number of call attempts, the number of successful calls (as defined by the Boost NP speaking to a Veteran), the number of completed encounters, the number and type of referrals ordered, the number and type of diagnostics ordered, if medication management such as refills or titration occurred, any additional care coordination activities such as calling consultants, if the Veteran needed assistance arranging VHA primary care follow-up, and overall Veteran satisfaction with the Boost outreach call were tracked on a de-identified secure data source. Basic demographics (age, gender identity, rurality, and service era) were collected from CDW (Figure 2).

Call conversion was calculated by dividing the total number of unique Veterans called by the total number of unique Veterans who answered the call and agreed to receive the Boost outreach intervention. Actions taken during the outreach encounter including number and type of referrals ordered, number and type of diagnostics ordered, if medication management was done, all specialty care coordination activities, and number of requests for assistance coordinating primary care follow-up were all tallied and reported both as sums and as percentages by dividing the sums of each intervention activity by the number of successful unique calls. At the end of each call, Veterans were invited to share feedback about the outreach experience. Qualitative data were not systematically analyzed and instead used to help iteratively inform process improvement and guide future program evaluation efforts.

## Results

### Needs assessment

The needs assessment demonstrated educational gaps for Veterans, staff, administrators, and clinicians, and a lack of gender-sensitive and women's-specific service offerings for rural women Veterans in VISN 21 (Figure 2). Although staff, administrators, clinicians, and Veterans represent disparate cohorts, all groups expressed similar concerns across all Precede-Proceed domains (8).

In the social assessment domain, multiple respondents reported that women Veterans do not identify as “Veterans”, and many cited staffing shortages at rural clinics as the cause of delays-in-care, low morale, and barriers to piloting new programing aimed at expanding services. In the epidemiologic domain, many reported that rural clinicians see a low volume of women Veterans, leading to women feeling uncomfortable that they are receiving outdated care. Similarly, interviewees noted knowledge gaps about women's health service offerings. In the behavioral and environmental domains, respondents discussed concerns about mixed-gender waiting rooms and shuttles, and many reported either personally seeing or hearing of unwanted or inappropriate comments made to female-presenting Veterans. In the educational and ecologic domains, many brought up a lack of trauma-informed care practices, and similar to the epidemiologic domain, discussed concerns about knowledge gaps related to women's health, as well as VHA gender-specific service-offerings. All interviewees commented that VHA is a challenging environment to navigate and many went into detail about system-level challenges that make accessing and utilizing care difficult. Lastly, in the administrative and policy domain, many reported feeling unease about information disclosure to VHA and frustration and confusion about community care and care coordination concerns.

### Outreach intervention: feasibility testing/pilot

**Figure 3:**
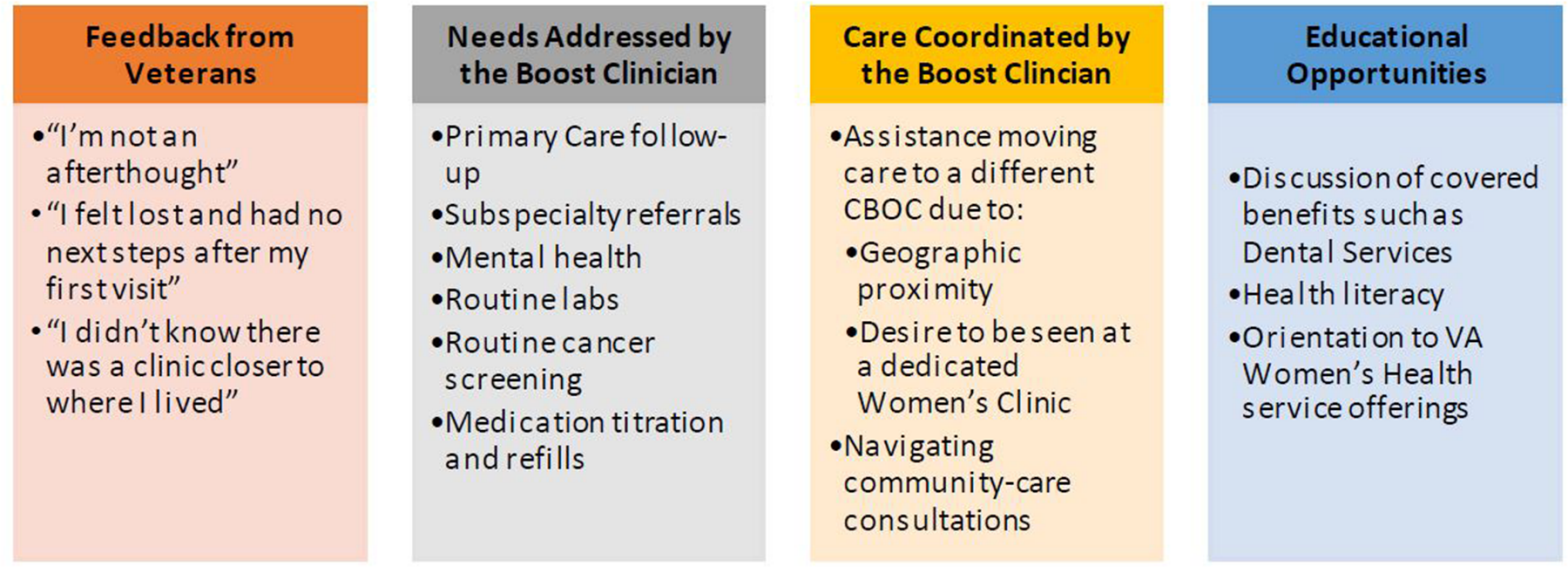
Lessons from the field: formative data from pilot outreach calls. *12 call attempts were made, 7 women were reached, 1 number was out of service, and 4 calls went to voicemail.

During our pilot in March 2022, 58% (7/12) rural cisgender women Veterans were successfully reached, and 100% (7/7) accepted outreach services. Of those, 100% (*n* = 7) reported positive experiences with the outreach call. Additionally, 100% (*n* = 7) had at least one care need met during the call: 86% (*n* = 6) had at least one referral placed, and 100% (*n* = 7) requested assistance arranging follow-up (Figure 3).

### Outreach intervention: PDSA cycle 1

**Figure 4:**
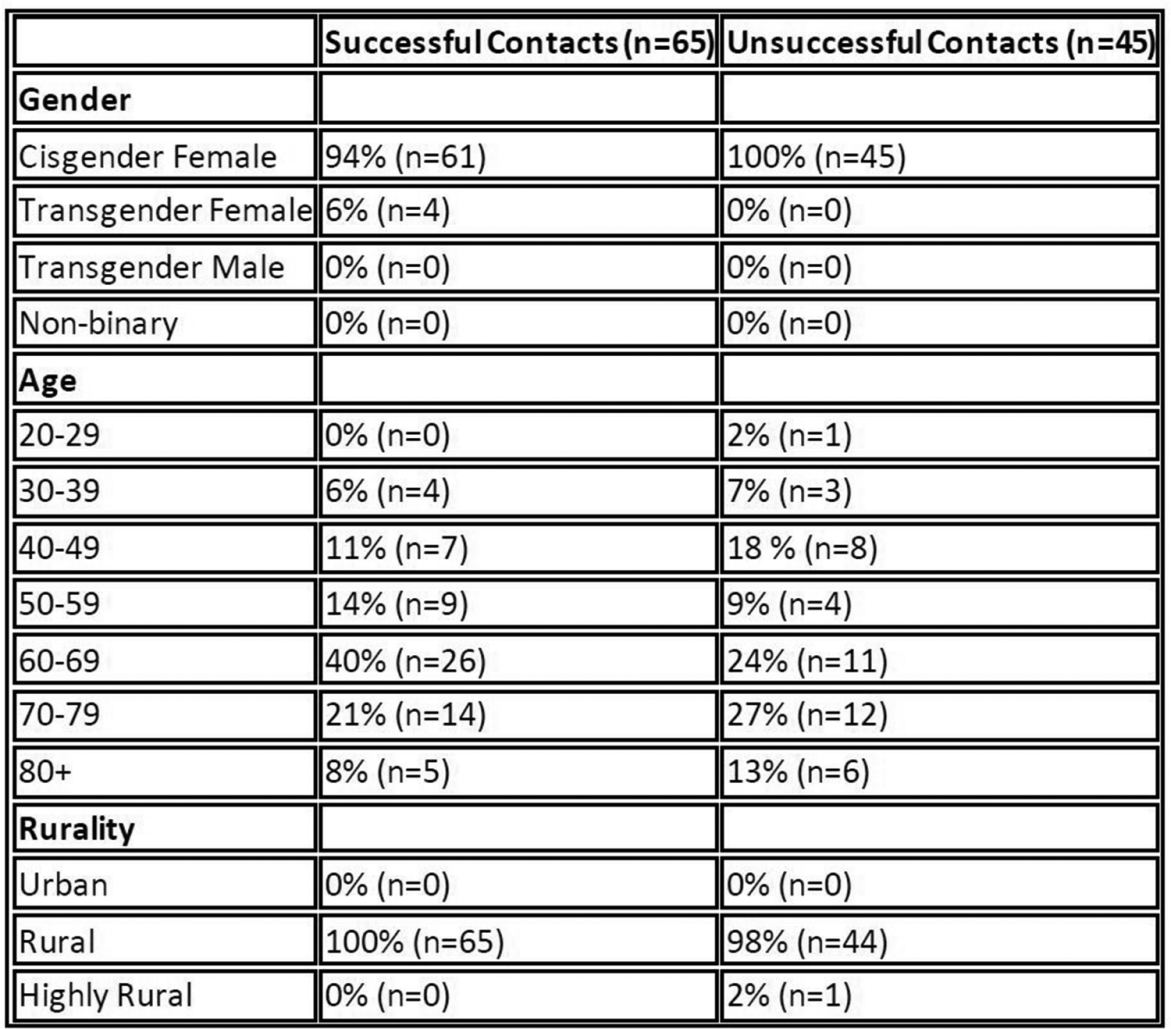
Demographics of Veterans called during PDSA Cycle 1. Of note: urban, rural, and highly rural, as defined by the VHA urban/rural/highly rural (URH) classification schema (9).

During PDSA Cycle 1, which ran from mid-September 2022 to December 2022, the Boost NP called 110 rural cisgender women and gender diverse Veterans and spoke to 65 of them (Figure 4). All Veterans (65/65) accepted outreach services and the Boost NP was able to address at least one health-related concern during the call. Common actionable care items included: 38% (*n* = 25) received assistance with care coordination with an established specialty service, 51% (*n* = 33) received one or more new referrals, 34% (*n* = 22) received assistance arranging follow-up with a VHA Primary Care Provider, 28% (*n* = 18) received assistance with medication management, and 6% (*n* = 4) received new laboratory orders. Veteran satisfaction data was uniformly positive and uncovered further opportunities to improve access to care (Figure 4).

## Discussion

### Implications

Our pilot and PDSA Cycle 1 of delivering the Boost intervention suggests that VHA clinician-led telephonic outreach efforts can engage rural cisgender women and gender diverse Veterans in VHA healthcare offerings and close health literacy and system-based practice gaps previously identified as barriers to using VHA care (10, 11). A key aspect of the Boost intervention is that calls are made by a clinician. Data from the needs assessment and outreach calls suggests that staff, administrators, clinicians, and Veterans harbor frustration and a loss of trust in the VHA owing to challenges in getting needs met. By having a clinician make the outreach calls, Veterans who received the Boost intervention were able to have their medical and psychosocial needs addressed in real-time.

Early data show that Boost's white-glove high-touch service can address unmet needs, such as specialty care coordination, and is greatly appreciated and valued by all stakeholder groups interviewed. Findings from the Boost intervention broadens support of the existing literature suggesting that telephone outreach to populations experiencing health disparities can improve engagement and close care gaps (3–5). Specifically, prior literature focused on the non-Veteran patient population and did not evaluate clinician-driven telephonic outreach; our work adds unique evidence supporting clinician-initiated outreach via telephone to rural cisgender women and gender diverse Veterans within the VHA setting. Based on initial findings, we hired a dedicated Boost clinician to continue clinical outreach calls, are creating a patient education campaign, and are developing a Boost Peer Support Specialist-run support groups focused on providing a sense of community to rural cisgender and gender diverse Veterans.

### Limitations

While initial findings were positive, our needs assessment was limited to one VISN, and the initial Boost outreach intervention was limited to one VHA facility focusing on rural cisgender women and gender diverse Veterans. Future evaluation is needed to determine how the intervention impacts rural cisgender women and gender diverse Veterans across different locations within the VHA, as well as how the intervention impacts other historically underserved and minoritized populations within the VHA.

### Future research

In order to iteratively improve the Boost intervention as well as ensure the intervention is achieving its goal of improving access to VHA care, we aim to create a robust program evaluation strategy using both qualitative and quantitative methods including semi-structured key stakeholder interviews that will be coded and analyzed, chart review to understand how the Boost intervention impacts care utilization patterns over time (including baseline pre-outreach call utilization as well as follow-through/engagement in care and utilization of VHA services at various time points after the outreach call), and Veteran surveys to ascertain how the intervention impacts engagement in and perceptions of VHA care. While the program is currently housed within one facility and focuses on cisgender women and gender diverse Veterans, we also hope to expand our geographic reach as well as pilot with other populations of under-served Veterans such as those in the broader lesbian, gay, bisexual, trangender, queer, plus + community.

## Conclusion

While clinician-driven outreach requires an upfront investment, preliminary data show that clinician-driven outreach is a powerful tool for restoring trust in the VHA, linking Veterans back to VHA care, and uncovering system-level opportunities to improve access to VHA care for rural cisgender women and gender diverse Veterans. Furthermore, initial data show that clinician-driven outreach can positively impact high-priority VHA primary care quality metrics, such as cancer screening, utilization of VHA services, and improved wellness of cisgender women and gender diverse Veterans. Although current efforts are focused on the broader San Francisco area, we aim to disseminate our findings broadly to improve access to gender-specific healthcare services among rural cisgender women and gender diverse Veterans throughout the United States. As part of our ongoing work, we will continue to collect and analyze qualitative and quantitative data from health system leaders, rural CBOC staff, and Veterans who received the Boost intervention utilizing individual interviews, electronic medical record chart review, and a validated survey tool measuring patient empowerment, engagement, and activation (12). Through rigorous program evaluation, we will iteratively optimize clinician-driven outreach to specific underserved populations and ideally create a national program aimed at serving more historically marginalized and underserved Veterans and illuminating opportunities for structural changes to improve access and decrease barriers to VHA care.

## Data Availability

The data that support the findings of this study are available from the corresponding author upon reasonable request.
